# Development, Characterization, and Evaluation of Topical Antibacterial Formulations From *Achyranthes aspera*

**DOI:** 10.1155/bmri/4493916

**Published:** 2026-03-31

**Authors:** Yohannes Reda Tesfay, Helen Bitew, Fantahun Molla Kassa, Tsadkan Gebremeskel Haile, Huria Hussien Fitwi, Afework Getachew Tadesse, Amanuel Teklebrhan Beyene, Desta Tesfay Mezgebo

**Affiliations:** ^1^ Department of Pharmaceutics, School of Pharmacy, College of Health Sciences, Mekelle University, Mekelle, Ethiopia, mu.edu.et; ^2^ Department of Pharmacognosy, School of Pharmacy, College of Health Sciences, Mekelle University, Mekelle, Ethiopia, mu.edu.et; ^3^ Department of Pharmaceutics and Social Pharmacy, College of Health Sciences, Addis Ababa University, Addis Ababa, Ethiopia, aau.edu.et

**Keywords:** *Achyranthes aspera*, herbal preparations, skin infections, *Staphylococcus aureus*, topical formulations

## Abstract

Skin diseases are a significant global health issue, particularly in developing nations. However, the efficiency of topical antibacterial treatments is diminishing due to the development of resistance. This study is aimed at developing and evaluating topical antibacterial formulations from *Achyranthes aspera*, a plant traditionally used for wound healing. The Kirby–Bauer method was employed to examine the antibacterial activity of hydroalcoholic extracts obtained from fresh and dried leaves of *A. aspera*. The fraction, which demonstrated the highest antibacterial activity, underwent further testing to determine its minimum inhibitory concentration (MIC) and was employed to formulate eight different topical formulations. These formulations were subsequently evaluated for their antibacterial effectiveness, sensory properties, pH, viscosity, spreadability, and acute dermal toxicity and underwent FTIR compatibility analysis and 3‐month accelerated stability studies (40°C/75% RH). The results indicated that the 70% hydroalcoholic extracts of *A. aspera* were effective against common skin bacteria, particularly *Staphylococcus aureus*. Among the fractions tested, the chloroform fraction exhibited the largest zones of inhibition (16.50 ± 0.76 mm for *S. aureus* and 15.33 ± 0.33 mm for *Pseudomonas aeruginosa*) and an MIC of 3.125 mg/mL for both bacteria. Among the eight formulations, the basic cream demonstrated the highest antibacterial performance with a ZI of 19 ± 0.577, followed by the emulsifying ointments. FTIR analysis confirmed the absence of chemical interactions between the active fraction and formulation excipients. All formulations displayed favorable pH levels, good spreadability, homogeneity, and stability and did not cause irritation when applied to mice at a dosage of 2000 mg/kg. The accelerated stability study confirmed that the basic cream and emulsifying ointment maintained their physicochemical properties and antibacterial efficacy over 3 months. The findings suggest that *A. aspera* chloroform fractions are promising for developing effective topical treatments for *S*. *aureus*–related skin infections.

## 1. Introduction

The skin, the body′s largest organ, serves as a dynamic barrier protecting against environmental, microbial, and chemical insults. In addition to its immunological and thermoregulatory functions, it plays a critical role in maintaining homeostasis [1]. Despite these defenses, it is highly susceptible to infections, inflammatory diseases, and neglected tropical skin conditions [2].

Skin diseases represent a significant concern in public health, affecting approximately one‐third of the global population and ranking as the fourth most prevalent health condition among individuals [2], yet they remain underprioritized in primary healthcare systems, especially in low‐ and middle‐income countries [3]. In Ethiopia, the burden is evident, with common conditions such as impetigo (16.3%), scabies (13.8%), and eczema (11.4%) being highly prevalent [4]. Similarly, 23.9% of eczematous dermatitis cases involve bacterial or fungal infections and infestations, complicating treatment and increasing the risk of antimicrobial resistance [5]. Comparable findings were also reported by Monari et al. [6], indicating that 21.5% of patients visiting a dermatology clinic in Northern Ethiopia presented with infection‐related skin diseases. On the global scale, skin diseases were the 10^th^ and 12^th^ leading nonfatal conditions for females and males, respectively, in 2017 [7], with their prevalence rising from 3.9% to 4.7% over the previous decade [8], underscoring the need for scalable and effective interventions.

Among skin‐related health issues, skin and soft tissue infections (SSTIs) are a significant concern due to their wide clinical spectrum and high prevalence, especially in developing regions, contributing to nearly 10% of hospital admissions [9]. SSTIs encompass both superficial and deep infections caused primarily by bacteria, particularly *Staphylococcus aureus* and *Streptococcus pyogenes*, and range from mild conditions such as impetigo and folliculitis to serious, invasive diseases like cellulitis and necrotizing fasciitis. The emergence and spread of antimicrobial‐resistant strains, especially methicillin‐resistant *S. aureus* (MRSA) and multidrug‐resistant *Pseudomonas aeruginosa*, have further complicated SSTI management and highlighted the limitations of conventional treatments [10, 11].

In this context, medicinal plants with established ethnopharmacological uses offer promising alternatives for antimicrobial therapy. Medicinal plants have gained renewed interest as sources of bioactive compounds with antimicrobial, anti‐inflammatory, and wound‐healing properties [12]. One such plant is *Achyranthes aspera* L. (Amaranthaceae), known locally in Ethiopia as “Mechelo” or “Telenj.” It has long been used in traditional medicine to treat wounds, infections, and inflammatory skin conditions [13–15]. Phytochemical investigations have identified a wide range of active constituents in *A. aspera*, including saponins (e.g., oleanolic acid), alkaloids (e.g., achyranthine), and phenolic compounds such as rutin and chlorogenic acid [16, 17]. Several studies have reported its antimicrobial potential against clinically relevant bacterial strains ([18]; P. [19, 20]).

Topical semisolid formulations such as creams and ointments offer targeted drug delivery, reduced systemic exposure, and improved patient compliance [21]. However, the development of such formulations from plant extracts involves key challenges, particularly concerning the selection of appropriate bases and excipients that support stability and bioactivity. Factors such as the solubility of phytochemicals, base–extract interactions, emulsifier presence, and physicochemical parameters such as pH, viscosity, and spreadability play critical roles in determining both therapeutic efficacy and formulation stability [22–24]. Despite existing reports on the antibacterial properties of *A. aspera*, there is a lack of comprehensive studies exploring its formulation into stable and effective topical preparations.

Therefore, the present study is aimed at developing and evaluating topical semisolid formulations prepared from the leaves of *A. aspera*. Specifically, we sought to assess their physicochemical properties, antimicrobial activity against common SSTI‐causing bacteria, and the influence of formulation variables on performance and stability. This work is aimed at bridging the gap between ethnobotanical knowledge and modern pharmaceutical formulation approaches for treating skin infections.

## 2. Materials and Methods

### 2.1. Chemicals and Reagents

BI‐distilled water (Jouri Labs, Addis Ababa, Ethiopia), 97% *v*/*v* ethanol (Kassa Manufacturing Company, Addis Ababa, Ethiopia), chloroform (Sisco Research Laboratories, New Mumbai, India), dimethyl sulfoxide (DMSO) 99% (Blulux Laboratories, India), hexane (Pentokey Organy, India), trimethylamine (HI Media Laboratories, Mumbai, India), emulsifying wax (Sisco Research Laboratories, Maharashtra, India), resazurin sodium salt (Thermo Fisher Scientific, Lancashire, United Kingdom), potassium hydroxide (Techno Pharmchem, Bhadurgarh, India), and stearic acid (Techno Pharmchem, Bhadurgarh, India) were obtained from their respective vendors. The Addis Pharmaceutical Factory (Adigrat, Ethiopia) generously gifted carbomer 934 (batch no.: 22113022), propylene glycol (batch no.: HXY/SD1806), and white soft paraffin (WSPB 1057116‐17). The chemicals and reagents used were of analytical grade.

### 2.2. Media and Reference Standard

Mueller–Hinton agar (HI Media Laboratories, Mumbai, India), Mueller–Hinton broth (Tulip Diagnostics, Verna, India), and nutrient agar (Tulip Diagnostics, Verna, India) were purchased from their respective vendors. The Microbiology Department of Mekelle University generously gifted ciprofloxacin discs (5 *μ*g).

### 2.3. Experimental Animals and Ethical Clearance

Female albino mice weighing 29–34 g were obtained from the animal house at Mekelle University′s College of Health Sciences School of Pharmacy, Pharmacology and Toxicology Department. The Health Research Ethics Review Committee of Mekelle University College of Health Sciences granted ethical approval under the registration number ERC 1664/2020.

### 2.4. Test Strains

Four different types of bacteria were used in the experiment, including two Gram‐negative bacteria, *Escherichia coli* (ATCC 25922) and *P. aeruginosa* (ATCC 27853), and two Gram‐positive bacteria, *S. aureus* (ATCC 25923) and *S. pyogenes* (ATCC 919120). All American Type Culture Collections were obtained from the Department of Hospital Microbiology at Ayder Comprehensive Specialized Hospital.

### 2.5. Statistical Analysis

Analysis was executed using SPSS Version 26 with one‐way ANOVA followed by Tukey′s post hoc test. Results were expressed as mean value ± standard error of the mean (SEM) and standard deviation (SD); *p* values less than 0.05 were considered significant.

## 3. Methods

### 3.1. Collection, Identification, and Preparation of Plant Material

The leaves of *A. aspera* were collected from May Mekden in the Tigray Region of Ethiopia in December 2019 (geographical coordinates: 13°34 ^′^58 ^″^ north and 39°34 ^′^05 ^″^ east). The plant specimen was identified and authenticated by Melaku Wendaferash at the National Herbarium of Addis Ababa University, where a voucher specimen (voucher no.: YR001) was deposited for reference. The collected leaves were then washed with clean water, shade‐dried to preserve their bioactive constituents, and ground into a coarse powder using a coffee bean grinder for further analysis.

### 3.2. Preparation of Crude Extracts

Extraction of plant materials was performed using a standard maceration technique. To prepare the samples, 500 g of fresh leaves and 500 g of the previously dried and powdered leaves of *A*. *aspera* were separately soaked in 70% ethanol mixed with water at a ratio of 1:7 *v*/*v*. A hydroalcoholic solvent (70% *v*/*v* ethanol) was selected for the extraction due to its proven efficacy in extracting a broad spectrum of medium‐ to high‐polarity bioactive compounds from plant materials, such as phenolics, flavonoids, and saponins [25]. These compound classes are frequently associated with antimicrobial activity, making this solvent appropriate for the aims of this study. The mixture was shaken twice daily for 3 days at 120 rpm using an orbital shaker for 30 min per agitation. After 72 h, the macerate was filtered using a muslin cloth and Whatman No. 1 filter paper with the assistance of a vacuum suction pump. The residue was then remacerated twice with a fresh solvent and filtered in the same manner. The filtrate was dried in an oven at 40°C until the solvent was completely evaporated. The resulting crude material was powdered using a mortar and pestle and stored in a glass container wrapped with aluminum foil in a refrigerator for subsequent use.

### 3.3. Preparation of Fractions

The crude extracts of *A*. *aspera* were further partitioned/fractionated using solvents with different polarity indices (*n*‐hexane, chloroform, and water) to concentrate secondary metabolites with the highest antibacterial activity in one fraction.

The fractionation procedure was adapted from Sarker et al. [26], who described a classical liquid–liquid extraction technique for the isolation of phytoconstituents based on differential solubility in immiscible solvents. Briefly, crude hydroalcoholic extracts of fresh *A*. *aspera* leaves (50 g) were dissolved in 500 mL of distilled water in a separatory funnel. An equal volume of hexane (500 mL) was added, and the mixture was vigorously shaken for 5 min to ensure thorough partitioning. After shaking, the mixture was allowed to settle until complete separation of the layers was achieved (~30 min). The aqueous layer (bottom phase) was carefully eluted and retained, while the hexane layer (top phase) was collected separately. This hexane extraction process was repeated twice more (3× total) with fresh 500 mL aliquots of hexane each time, and all hexane filtrates were pooled for consistency.

The remaining aqueous filtrate was then subjected to chloroform fractionation using the same protocol: An equal volume of chloroform (500 mL) was added, followed by vigorous shaking, phase separation, and collection of the lower chloroform layer. This step was also repeated twice (three times in total) with fresh 500 mL chloroform aliquots, and all chloroform filtrates were pooled.

Finally, the aqueous residue, the pooled chloroform fraction, and the pooled hexane fraction were dried separately in an oven at 40°C until they become dry. The percentage yield of each fraction was calculated using the following equation:

Percentage yield=amount g of the dried crude extract obtainedamount g of grounded plant material used×100.

### 3.4. Antibacterial Activity Screening

The antibacterial activity was evaluated using a modified Kirby–Bauer disk diffusion method [27, 28]. The crude extracts were tested at three concentration levels (200, 100, and 50 mg/mL), while fractionation was assessed only at 200 mg/mL. DMSO 2% *v*/*v* and 5 *μ*g ciprofloxacin served as the negative and positive controls, respectively. Each test was performed in triplicate, and the mean inhibition zone diameter (mm), including the discs, was measured using a ruler. Both the growth and sterility controls were conducted in parallel for every experiment. The detailed procedures for the antibacterial tests are listed below.

#### 3.4.1. Disc Diffusion Assay

A disc diffusion assay was conducted to evaluate the antimicrobial activity of plant extracts. To begin, sterilized agar medium (20 mL) was poured into sterile 90‐mm‐diameter Petri dishes in an aseptic manner. The plates were then allowed to solidify at room temperature within a Class II microbiological safety cabinet (Labcaire). After an incubation period of 16–18 h at 37°C in a Kendro incubator, the Petri dishes were visually inspected for microbial growth to ensure sterility, as per Salie et al. [28].

For inoculation, a sterile cotton swab was dipped into a previously prepared bacterial suspension and rotated to ensure even distribution of the bacteria. The swab was then gently pressed against the test tube wall to remove excess inoculum. Using a presterilized cotton swab at a 45° angle, the respective bacteria were streaked onto the solidified agar medium, with the swab being rotated at least three times to ensure complete coverage and a uniform growth pattern, following the methods of Messele et al. [29] and Tadeg et al. [30].

Simultaneously, paper discs with a diameter of 6 mm were prepared by punching Whatman No. 1 filter paper. These discs were then sterilized under UV light for 1 h. A predetermined amount of plant extract at different concentrations (20 *μ*L of 200, 100, and 50 mg suspended in 2% *v*/*v* DMSO) was loaded onto each disc, resulting in final concentrations of 4, 2, and 1 mg per disc, respectively. After allowing the impregnated discs to dry for 15 min under lamellar airflow in the safety cabinet, they were carefully placed onto the agar plates using sterile forceps. To maintain equidistance, the discs were spaced apart from each other, with the middle disc serving as the positive control (5 *μ*g ciprofloxacin) and a negative control containing only the vehicle (2% *v*/*v* DMSO). DMSO at 2% *v*/*v* concentration was used as a vehicle to solubilize the plant extracts and fractions due to its ability to dissolve both polar and nonpolar phytoconstituents, ensuring uniform loading onto the discs and facilitating diffusion into the aqueous agar medium. This concentration was selected because it is widely reported in the literature as nontoxic to bacteria and does not inhibit bacterial growth, as confirmed by the negative control (2% DMSO alone) showing no zone of inhibition against any of the test strains. This methodology aligns with the approaches described by Salie et al. [28].

Following disc placement, the Petri dishes were preincubated under lamellar airflow in the safety cabinet for 30 min to facilitate the diffusion of the extract into the agar medium. Subsequently, the dishes were inverted and incubated overnight at 37°C, ensuring that the caps were positioned at the bottom and the test materials were positioned at the top to prevent secondary contamination due to evaporation considerations, as recommended by EUCAST [31].

#### 3.4.2. Minimum Inhibitory Concentration (MIC) Determination Using the Broth Microdilution Assay

The fraction that showed the highest zone of inhibition in the disc diffusion assay was further investigated for the MIC using resazurin sodium as an indicator in a broth microdilution assay [31–33].

Initially, 100 *μ*L of Mueller–Hinton broth was dispensed into each well of a 96‐well microtiter plate using a multichannel micropipette. A 100 *μ*L aliquot of the test extract stock solution (100 mg/mL) was then added to the wells in Column 1 and mixed thoroughly using a multichannel dispenser. Twofold serial dilutions were performed by transferring 100 *μ*L from Column 1 to Column 2, and the process was repeated sequentially through Column 10. In Column 10, 100 *μ*L was discarded after mixing to maintain a consistent volume of 100 *μ*L per well, resulting in final extract concentrations ranging from 100 mg/mL (Column 1) to 0.195 mg/mL (Column 10) [32, 33].

Bacterial strains sensitive to the most active extract fraction were selected for MIC testing. A bacterial suspension was prepared by adjusting to a 0.5 McFarland standard, followed by a 1:100 dilution with 0.85% saline to yield a final concentration of 5 × 10^6^ CFU/mL. Then, 20 *μ*L of this suspension was added to each well (Columns 1–10), resulting in a total well volume of 120 *μ*L and a final bacterial concentration of approximately 8.33 × 10^5^ CFU/mL, which is higher than the EUCAST‐recommended standard of 1 × 10^5^ CFU/mL. This approach was adopted to maintain consistency with the methodology described by Asmerom et al. [31]; hence, this may influence absolute MIC values and their comparability with other studies using the standard inoculum.

Wells in Column 11 served as sterility controls (broth only), while Column 12 served as growth controls (broth and bacteria without test extract). The plates were loosely covered with aluminum foil to prevent evaporation and incubated at 37°C ± 2°C for 18–24 h [33–35].

After the 24‐h incubation period, bacterial viability was assessed using a resazurin‐based colorimetric assay. A 0.015% (*w*/*v*) resazurin sodium salt solution (prepared in sterile distilled water) was added (25 *μ*L per well) to all test columns (1–12) and incubated at 37°C for 2–3 h. Metabolic activity of viable bacteria reduces resazurin (blue/purple) to resorufin (pink), yielding a visual and spectrophotometric endpoint. The MIC was defined as the lowest test concentration showing no color change (blue/purple, indicating inhibition) compared to the pink of the growth control. To ensure reproducibility, the assay was repeated in triplicate, and the modal MIC value (most frequently observed result) was reported [33–35].

### 3.5. Preparation of Semisolid Bases

Eight semisolid bases were selected with the intention of covering a wide range of formulation types commonly used in topical preparations with a variety of polarity indices. These included oleaginous (simple ointment), absorption‐type (emulsifying ointment), water‐removable (hydrophilic ointment, vanishing cream, and basic cream), and polymeric gel systems of carbomer‐based (Carbopol 934) and cellulose derivative gels (sodium CMC and HPMC gels). These semisolid bases, classified as ointments, gels, or creams, were prepared according to standard methods (Aiyalu, Govindarjan, and Ramasamy 2016, Marriott et al. 2010). Their compositions are detailed in Tables 1, 2, and 3.

**Table 1:** Ointment bases.

Ointments	Emulsifying wax (g)	White soft paraffin (g)	Liquid paraffin (mL)	Cetostearyl alcohol (g)	Hard paraffin (g)	Wool fat (g)	Sodium lauryl sulfate (g)	Purified water (mL)
Emulsifying	30	45	25	—	—	—	—	—
Hydrophilic	—	40	—	16	—	—	1	To 100
Simple	—	85	—	5	5	5	—	—

**Table 2:** Gel bases.

Gels	Carbopol 934 (mg)	HPMC (g)	NaCMC (g)	Propylene glycol (mL)	Triethanolamine (mL)	Purified water (mL)
HPMC	—	1.5	—	1.5	—	To 15
CMC	—	—	0.45	1.5	—	To 15
Carbopol	0.15	—	—	1.5	2	To 15

**Table 3:** Cream bases.

Creams	Emulsifying wax (g)	Glycerol (mL)	Liquid paraffin (mL)	Stearic acid (g)	Petrolatum (g)	KOH (g)	Purified water (mL)
Basic	15		12.5		22.5		To 100
Vanishing		5		15		1	To 100

Among these, three formulations are official in pharmacopeias: simple ointment (British Pharmacopoeia and US Pharmacopeia), emulsifying ointment (British Pharmacopoeia), and hydrophilic ointment (US Pharmacopeia). These were prepared exactly according to the official formulas using the fusion method, with components combined in an evaporating dish and melted together over a water bath following the order of their decreasing melting points [37].

Ointments (Table 1) were prepared by the fusion method. Components were combined in an evaporating dish and melted together over a water bath, following the order of their decreasing melting points [37].

On the other hand, gels (Table 2) were prepared by dispersing the gelling agent (Carbopol 934, HPMC, or NaCMC) in purified water. Propylene glycol was incorporated, and the mixture was stirred and allowed to hydrate overnight. For the carbopol gel, triethanolamine was added last to neutralize the mixture and form a clear gel [36].

Creams (Table 3) were prepared by emulsification. The oil‐soluble and water‐soluble components were heated separately to 60°C–75°C. The aqueous phase was then added to the oil phase with continuous stirring until the mixture cooled and a homogeneous cream consistency was achieved [37].

### 3.6. Incorporation of Extracts in Semisolid Bases

Eight topical semisolid formulations were prepared using the most active solvent fraction. The formulations were adjusted to a concentration of 10%, with 1 g of the extract added to 9 g of semisolid base through trituration/levigation and fusion methods. The extract was incorporated into the formulation base at a concentration of 10% *w*/*w*. This concentration was selected based on its common use and demonstrated efficacy in prior phytopharmaceutical studies of topical formulations [29, 30]. Furthermore, this concentration ensures a significant payload of extract (100 mg/g) to overcome the inefficient release kinetics from ointment bases and effectively deliver the active compounds at a concentration surpassing the determined MIC value of the extract, creating a sink condition so as to keep a concentration gradient. For insoluble solids, the powders were levigated in an ointment tile with a spatula until a homogeneous formulation was obtained. The ointments and gels were prepared using this method, with the extract levigated in one direction to prevent air incorporation. For soluble solids, the powders were added to molten and cold bases with stirring until cold, a process known as fusion. The powders soluble in cream bases were incorporated using the fusion method [37].

### 3.7. Chemical Compatibility Study Using Fourier Transform Infrared (FTIR) Spectroscopy

FTIR spectroscopy was conducted at Addis Ababa Science and Technology University to evaluate the potential chemical interactions between the active fraction (*A. aspera* hexane fraction) and the commonly used pharmaceutical excipients in the cream formulations. The spectra of the pure *A*. *aspera* hexane fraction, the placebo formulations (basic cream only and emulsifying ointment only), and the corresponding medicated formulations (basic cream with *A*. *aspera* fraction and emulsifying ointment with *A*. *aspera* fraction) were obtained and compared.

Samples were prepared using the potassium bromide (KBr) pellet method. Each sample was thoroughly mixed with dry KBr powder and compressed into a transparent disc. The FTIR spectra were recorded over a wavenumber range of 4000–400 cm^−1^ using an FTIR spectrometer. The characteristic absorption peaks of the pure fraction and the placebos were identified and compared with the peaks observed in the spectra of the final cream formulations. A shift in the position of characteristic peaks or the appearance/disappearance of peaks in the formulation spectra would indicate a potential physicochemical interaction between the fraction and the excipients.

### 3.8. Antibacterial Performance Testing of Topical Formulations

The antibacterial activity of the prepared formulations was assessed using Nathan′s agar well diffusion (NAWD) (Nathan et al. 1978) method with slight modifications [30, 39, 40]. A sterile Petri dish (90 mm in diameter) was filled with 15 mL of molten and cooled agar, creating a solid surface. Equidistant holes, 10 mm in diameter, were made using a sterile cork borer or tip. The agar cut‐outs were then removed using a sterile needle. Each well was filled with 0.1 mL, equivalent to 0.2 g of the designated topical agents, using sterile 3‐mL syringes. Next, 0.2 mL of the overnight cultured bacterial suspension in nutrient broth was added to 7 mL of molten and cooled agar and mixed thoroughly using a vortex mixer. This solution was poured onto the solid agar, sandwiching the formulation. After solidification for 2–3 min, the plates were inverted and left at room temperature for 1 h, followed by incubation at 37°C for 24 h. The diameter of the zone of inhibition was measured immediately after incubation using a ruler to the nearest millimeter.

### 3.9. Evaluation of Topical Formulations

The formulations that demonstrated significant antibacterial activity were further evaluated for the following parameters. Each test was conducted in triplicate, and the results were reported as mean ± SD.

#### 3.9.1. Sensory and Tactile Evaluation

A panel of 10 trained individuals, consisting of five males and five females aged over 18 years, participated in the sensory and tactile evaluation of the semisolid preparations. Participants were recruited through purposive sampling at Mekelle University, Ethiopia, and were trained in accordance with the guidelines outlined in ISO 8586: 2012, which provides standardized procedures for the selection, training, and monitoring of sensory assessors [41]. A standardized 5‐point descriptive scale was used to assess the physical characteristics of the formulations, where 1 = *very poor*, 2 = *poor*, 3 = *fair*, 4 = *good*, and 5 = *excellent*. This approach is consistent with sensory analysis practices in topical and cosmetic product evaluation (M. [42]).

The evaluation focused on visual and tactile parameters, including appearance, color, texture, phase separation, and homogeneity. Texture and homogeneity were assessed by pressing approximately 0.5 g of each formulation between the thumb and index finger, as commonly practiced in the assessment of semisolid and emulsion‐based products. Each parameter was scored individually by all panelists, and the mean score for each attribute was calculated to represent the final evaluation for each formulation. Sensory data were analyzed using a linear mixed model in SPSS Version 26. The model specified product as a fixed effect and panelists as a random effect to account for individual variation in scale use. The significance level for all tests was set a priori at *α* = 0.05.

#### 3.9.2. Centrifugation

To assess phase separation and solid sedimentation, 10 g portions of each formulation were subjected to centrifugation at 3000 rpm for 30 min using a tabletop centrifuge (PLC‐03, Taiwan). Then phase separation and solid sedimentation were visually evaluated, as described by Gemeda et al. [40].

#### 3.9.3. Thermal Cycle Test

For the thermal cycle test, 5 g of the formulation was stored at 50°C for 48 h and then at 25°C for an additional 48 h. The physical stability and appearance of the samples were assessed visually after six repetitions [40].

#### 3.9.4. Determination of pH

To determine the pH, 1 g of the test formulation was dispersed in 25 mL of deionized water, and the pH was measured using a pH meter (Adwa, Szeged, Hungary). The glass electrode was immersed safely into the formulation, ensuring its complete coverage, while stirring with a magnetic stirrer. Tests were conducted in triplicates (Aiyalu et al., 2016, and Chen et al., 2016). Triplicate measurements were conducted, and the average value was determined according to the methods described by Aiyalu et al. [36] and Chen et al. [43].

#### 3.9.5. Spreadability Measurement

Spreadability measurements were conducted using the method described by Chen et al. [43] with slight modifications. A weight of 25 g was applied to 1 g of formulation that was spread on glass slides measuring 20 × 20 cm. After 1 min, the spreading diameter was recorded.

#### 3.9.6. Determination of Viscosity

Viscosity of the topical formulations was determined using a Brookfield Viscometer RV (Brookfield Engineering Laboratories, Middleboro, Massachusetts). The viscosity measurements were performed using Spindle No. 7 at rotational speeds of 12, 20, 30, and 50 rpm at a temperature of 21°C. Prior to measurement, the samples were equilibrated to the test temperature, following the method described by Chen et al. [43].

#### 3.9.7. Acute Dermal Toxicity Study

Acute dermal toxicity testing was conducted in accordance with the OECD Guideline No. 402 [44] after obtaining ethical approval from the Health Research Ethics Review Committee of Mekelle University College of Health Sciences (ERC 1664/2020). The animals were individually housed with free access to pellets and tap water, maintained under a 12‐h light/dark cycle at an ambient temperature of 22°C ± 3°C in compliance with institutional animal care standards. Prior to the experiment, a 5‐day acclimatization period was conducted, and a visual examination was conducted to ensure the absence of visible skin lesions. Female mice with normal skin texture were selected randomly and divided into test and control groups, each consisting of five mice. The mice were individually housed and allowed to acclimate for 1 week in their respective cages. Afterward, the mice were anesthetized with intraperitoneal ketamine (50 mg/kg), and 10% of the fur on the dorsal side of their trunk was shaved 24 h prior to the study. A semisolid preparation formulated with chloroform fraction at a dose of 2000 mg/kg (10% *v*/*v*) was uniformly applied and left on the shaved area for 24 h. Following the removal of any remaining test material, the mice were observed daily for 14 days to detect any adverse skin reactions, such as erythema and edema. These reactions were then evaluated and graded according to the OECD grading criteria. All procedures adhered to institutional ethical guidelines for laboratory animal welfare.

#### 3.9.8. Accelerated Stability Study

An accelerated stability study was conducted at the quality control laboratory of Addis Pharmaceutical Factory (Adigrat, Ethiopia) following ICH Q1A (R2) guidelines [45]. The two most promising formulations (basic cream and emulsifying ointment) were packaged in collapsible aluminum tubes and stored in a stability chamber maintained at 40°C ± 2°C and 75*%* ± 5*%* relative humidity for 3 months. Samples were withdrawn at predetermined intervals (0, 1, 2, and 3 months) and evaluated for physical appearance, pH, viscosity, spreadability, and antibacterial activity against *S. aureus* using the NAWD method. All tests were performed in triplicate, and a formulation was considered stable if no statistically significant changes ( ^∗^ *p* < 0.05) were observed in the evaluated parameters over the study period.

## 4. Results

### 4.1. Percentage of Yield and Antibacterial Activity of Crude Extracts

The percentage yields of crude extracts of *A*. *aspera*′s dried and fresh leaves were found to be 14.5% and 11.7%, respectively.

The results of the preliminary antibacterial screening assay are presented in Table 4. According to the table, all the 70% ethanol extracts showed antibacterial activity against most of the test organisms: A zone of inhibition of 10 or greater is considered promising for antibacterial activity. All strains were sensitive to the positive control and to some crude extracts in a concentration‐dependent manner.

**Table 4:** Zone of inhibition of the 70% (*v*/*v*) ethanol extracts of *Achyranthes aspera* leaves at different concentrations against bacterial strains.

Bacterial strains	Zone of inhibition (mm)						
	*Achyranthes aspera*						Cipro (*μ*g)
	Fresh extract (mg/mL)			Dry extract (mg/mL)			
	200	100	50	200	100	50	5
*E*. *coli*	8.3 ± 0.05^∗^^e^	7.73 ± 0.14^∗^^e^	7.4 ± 0.4	7.83 ± 0.16^∗^^e^	7 ± 0.57	6.33 ± 0.16	20
*P*. *aeruginosa*	14.16 ± 0.44^∗^^abcde^	12.50 ± 0.28^∗^^be^	10.16 ± 0.44^∗^^cd^	12.5 ± 0.28^∗^^e^	12.8 ± 0.06^∗^^e^	9.30 ± 0.44	32
*S*. *aureus*	16.60 ± 0.10^∗^^abcde^	14.50 ± 0.28	12.83 ± 0.44^∗^^c^	14.83 ± 0.16	14.23 ± 0.25	13.40 ± 0.17	31
*S*. *pyogenes*	15.4 ± 0.08^∗^^bde^	14.33 ± 0.08^∗^^e^	13.4 ± 0.03	14.36 ± 0.08^∗^^e^	13.48 ± 0.04	12.83 ± 0.16	31

*Note:* Values are expressed as mean ± SEM (*n* = 3). Analysis was carried out by SPSS Version 26 with one‐way ANOVA followed by Tukey′s post hoc test. ZI includes the zone of diameter of the disc = 6 mm. Antibacterial activity was determined using a modified Kirby diffusion assay.

^a^Compared with fresh 100 mg/mL.

^b^Compared with fresh 50 mg/mL.

^c^Compared with dry 200 mg/mL.

^d^Compared with dry 100 mg/mL.

^e^Compared with dry 50 mg/mL.

^∗^ *p* < 0.05.

The mean zone of inhibition differed significantly between the dry and fresh plant extracts. Fresh *A. aspera* leaves showed the highest inhibition zone (16.5 mm at 200 mg/mL) against *S. aureus*, followed by *S. pyogenes* (15.4 mm), *P. aeruginosa* (14.16 mm), and *E. coli* (8.3 mm). Similarly, a significant difference in activity (*p* < 0.05) was observed between the fresh and dried leaf extracts against *S*. *aureus*. Furthermore, Gram‐positive bacteria, particularly *S. aureus*, were more susceptible than Gram‐negative bacteria like *E. coli*.

### 4.2. The Antimicrobial Activities of Fractionates Using the Kirby–Bauer Disc Diffusion

The percentage yields of the fractions increased as the polarity of the fractionating solvents increased: 15%, 30%, and 42% for *n*‐hexane, chloroform, and water, respectively. The fractions displayed varying levels of antibacterial activities, as shown in Table 5. Among the fractions, chloroform showed significantly higher (*p* < 0.05) zone of inhibition of 16.50 ± 0.76 against *S*. *aureus*, followed by hexane and water at zones of inhibition of 13.33 ± 0.3 and 14.33 ± 0.3, respectively. Moreover, among the bacterial strains tested, relatively higher antibacterial activity was observed against *S*. *aureus* in all fractions, with the chloroform fraction showing the highest activity, even compared to the hydroalcoholic crude extract at the same dose.

**Table 5:** Zone of inhibition of the solvent fractions of the fresh leaf crude extract of *Achyranthes aspera* at 200 mg/mL against bacterial strains.

Bacteria strains	Zone of inhibition (mm)				
	Fractions			Cipro 5 *μ*g	DMSO
	Chloroform	Water	Hexane		
*S*. *aureus*	16.50 ± 0.76^∗^^ab^	13.33 ± 0.33	14.33 ± 0.33	31.0 ± 0.0	—
*S*. *pyogen*	14.26 ± 0.37^∗^^a^	11.50 ± 0.28^∗^^b^	13.80 ± 0.20	30.0 ± 0.0	—
*P*. *aeruginosa*	15.33 ± 0.33^∗^^ab^	13.30 ± 0.05	13.80 ± 0.15	31.0 ± 0.0	—
*E. coli*	—	—	—	20.0 ± 0.0	—

*Note:* Values are expressed as mean ± SEM (*n* = 3). Analysis was carried out by SPSS Version 26 with one‐way ANOVA followed by Tukey′s post hoc test. –: indicates no antibacterial activity. ZI includes the zone of diameter of the disc = 6 mm. Antibacterial activity was determined using the modified Kirby diffusion assay.

^a^Compared with water.

^b^Compared with hexane.

^c^Compared with the positive control.

^∗^ *p* < 0.05.

### 4.3. MIC of Chloroform Fractions

The MIC value of the chloroform fraction was 3.125 mg/mL for both *S*. *aureus* and *P*. *aeruginosa*, which agrees with the initial preliminary antimicrobial screening test and fractionation results.

### 4.4. FTIR Spectroscopy Analysis

FTIR spectroscopy was employed to characterize the functional groups present in the *A. aspera* hexane fraction and to evaluate its compatibility with the selected cream bases. Compatibility is established when the characteristic absorption peaks of the active pharmaceutical ingredient (API) are preserved in the formulation without significant shifts, disappearance, or the emergence of new peaks, which would indicate physicochemical interactions. The FTIR spectra of the pure hexane fraction, placebo formulations (vehicle), and medicated formulations were analyzed and compared.

The FTIR spectrum of the *A*. *aspera* hexane fraction is presented in Figure 1. A broad and strong absorption band observed at 3345.96 cm^−1^ was attributed to O–H stretching vibrations, indicating the presence of phenolic compounds or alcohols. The prominent peaks at 2918.22 and 2850.09 cm^−1^ were assigned to asymmetric and symmetric C–H stretching of methylene (–CH_2_) groups, confirming the aliphatic nature of constituents extracted by the nonpolar hexane solvent, likely including terpenoids, steroids, or fatty acid derivatives.

Figure 1Overlay of FTIR spectra for (a) *A*. *aspera* hexane fraction, (b) basic cream only, and (c) basic cream containing the fraction.

**Figure:**
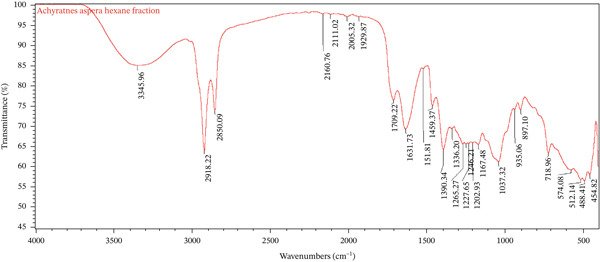
(a)

**Figure:**
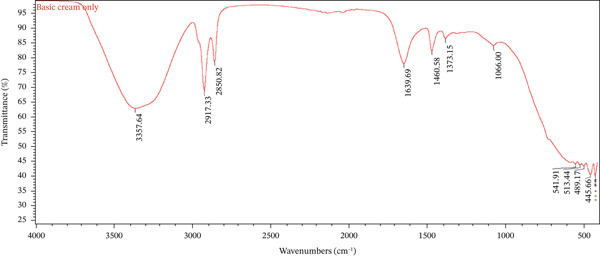
(b)

**Figure:**
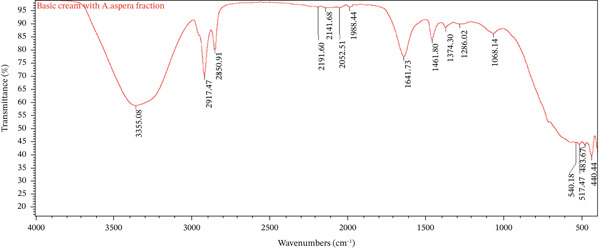
(c)

The absorption band at 1631.73 cm^−1^ corresponds to C=O stretching vibrations (amide or carbonyl compounds) or C=C stretching of aromatic rings. Additional peaks in the fingerprint region, including 1390.34 cm^−1^ (C–H bending), 1265.27 cm^−1^ (C–O stretching), and 1037.32 cm^−1^ (C–O–C stretching), further support the presence of diverse secondary metabolites such as flavonoids, saponins, and phytosterols.

The FTIR spectra of the placebo basic cream, *A*. *aspera* hexane fraction, and the medicated basic cream containing the fraction are overlaid in Figure 2. The placebo basic cream exhibited major absorption bands at 3357.64 cm^−1^ (O–H stretching), 2917.33 and 2850.82 cm^−1^ (C–H stretching), and 1639.69 cm^−1^ (C=O stretching), which are characteristic of the emulsifying and humectant components typically present in such bases (e.g., water, propylene glycol, and stearic acid).

Figure 2Overlay of FTIR spectra for (a) *A*. *aspera* hexane fraction, (b) emulsifying ointment only, and (c) emulsifying ointment containing the fraction.

**Figure:**
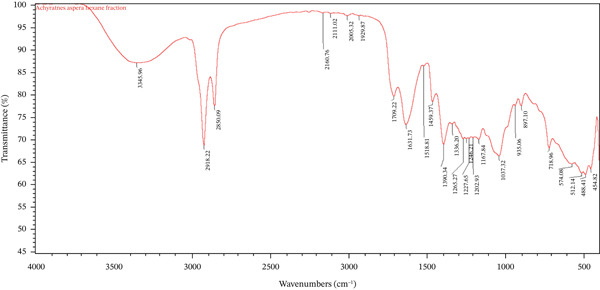
(a)

**Figure:**
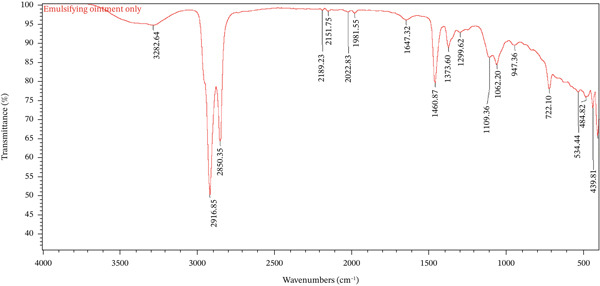
(b)

**Figure:**
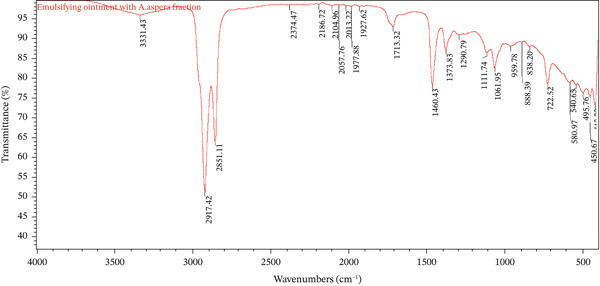
(c)

Upon incorporation of the *A*. *aspera* hexane fraction, the FTIR spectrum of the medicated basic cream showed all the major peaks corresponding to both the base and the API. The O–H stretching band appeared at 3355.08 cm^−1^, representing a minor shift (2.56 cm^−1^ from the base and 9.12 cm^−1^ from the API), which is within the acceptable range of instrumental or baseline variation. The aliphatic C–H stretching vibrations were observed at 2917.47 and 2850.91 cm^−1^, essentially unchanged from the placebo. Crucially, the characteristic fingerprint peaks of the API, such as those near 1631, 1390, and 1037 cm^−1^, were retained in the formulation without any significant shift or loss of intensity.

As clearly visualized in Figure 1, no new absorption bands were detected in the medicated formulation spectrum, and no characteristic peaks from either the base or the API disappeared. This observation confirms the absence of chemical interactions, such as hydrogen bonding changes, complexation, or degradation, between the *A*. *aspera* fraction and the components of the basic cream [34]. The formulation is therefore considered compatible at the molecular level.

**Figure 3:**
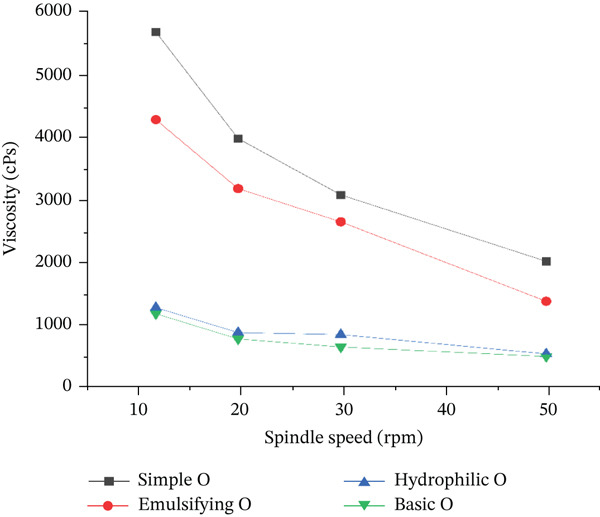
Viscosity curves for the selected semisolid preparations (*n* = 3, results shown as mean ± SD).

The FTIR spectra of the placebo emulsifying ointment, *A*. *aspera* hexane fraction, and the medicated emulsifying ointment are presented in Figure 3. The placebo emulsifying ointment exhibited characteristic peaks at 2928.64 and 2850.35 cm^−1^ (C–H stretching of long‐chain fatty alcohols and hydrocarbons) and a peak at 1647.32 cm^−1^ (C=O stretching). Examination of the raw spectral data confirmed the presence of the ester carbonyl peak around 1733 cm^−1^, which is characteristic of the ester components in emulsifying wax (e.g., cetostearyl alcohol/cetrimide wax) and serves as a critical diagnostic feature.

The spectrum of the medicated emulsifying ointment showed the presence of the API peaks superimposed on the base spectrum. The aliphatic C–H stretches appeared at 2917.42 and 2851.11 cm^−1^, consistent with the placebo. The ester carbonyl peak was clearly observed at 1713.32 cm^−1^, confirming its presence in the formulation and ruling out any interaction affecting this functional group. The API′s O–H band (originally at 3345.96 cm^−1^) is merged with the broad background of the ointment base, as can be observed in the overlay.

### 4.5. Antibacterial Activities of Semisolid Preparations Using NAWD

As shown in Table 6, four out of the eight tested formulations (basic cream, emulsifying ointment, simple ointment, and hydrophilic ointment) exhibited antibacterial activity against *S. aureus*, with average inhibition zones ranging from 12 to 19 mm. The basic cream demonstrated the highest activity among all formulations, with an average inhibition zone of 19 mm. Overall, the ointments showed significantly higher antibacterial activity compared to the creams and gels. Among them, emulsifying ointment was the most effective (16.6 mm), followed by simple ointment (15.1 mm) and hydrophilic ointment (12.1 mm). The positive control displayed significantly stronger activity than all formulations (*p* < 0.01).

**Table 6:** Zone of inhibition of 10% topical preparations from chloroform fraction of fresh leaves of *Achyranthes aspera* against *Staphylococcus aureus.*

Formulation	Conc. % *v*/*v*	Zone of inhibition (mm)
Emulsifying ointment	10	16.66 ± 0.33^∗^^acdeg^
Hydrophilic ointment	10	12.16 ± 0.166^∗^^bf^
Simple ointment	10	15.16 ± 0.166^∗^^cdefg^
CMC‐based gel	10	11.66 ± 1.66^∗^^f^
HPMC‐based gel	10	10.0 ± 0.00^∗^^f^
Basic cream	10	19 ± 0.577^∗^^g^
Vanishing cream	10	10.0 ± 0.00
TTC ointment	3	25.0 ± 0.00
Fusidic acid cream	2	37.0 ± 0.00

*Note:* Values are expressed as mean ± SEM (*n* = 3). Analysis was carried out by SPSS Version 26 with one‐way ANOVA followed by Tukey′s post hoc test. ZI includes the zone of diameter of the well = 10 mm. Antibacterial activity was determined using a modified Kirby diffusion assay.

Abbreviations: CMC, carboxymethyl cellulose; HPMC, hydroxypropyl methylcellulose; TTC, tetracycline.

^a^Compared to a hydrophilic ointment.

^b^Compared to a simple ointment.

^c^Compared with carbopol gel.

^d^Compared with CMC gel.

^e^Compared with HPMC gel.

^f^Compared with basic cream.

^g^Compared with vanishing cream.

^∗^ *p* < 0.05.

### 4.6. Acute Dermal Toxicity

The results of acute dermal toxicity tests of chloroform fractions of 70% ethanol fresh leaf extract of *A*. *aspera* indicate no sign of inflammation, irritation, or redness. Moreover, there were no gross behavioral changes or mortality within 24 h, as well as the next 14 days, during cage‐side observation. Additionally, Table 7 shows the weight change of mice throughout the study period. As shown in the table, the mice had no significant increase in weight throughout the study period.

**Table 7:** Effects of chloroform fractions of 70% ethanol fresh leaf extract of *Achyranthes aspera* on the body weight of mice at different days.

Group	Dose	Weight (g)		
		Initial day	7^th^ day	14^th^ day
I	Semisolid base	31.28 ± 0.72	32.74 ± 0.39	31.66 ± 0.53
II	2000 mg/kg	31.36 ± 0.61	33.38 ± 1.7	31.54 ± 2.93

*Note:* Values are expressed as mean ± SD (*n* = 5). Group I: Only a semisolid base was applied. Group II: 2000 mg/kg of chloroform fraction of *Achyranthes aspera* as a basic cream was applied.

### 4.7. Physicochemical and Sensory Characterization of the Semisolid Formulations

**Figure 4:**
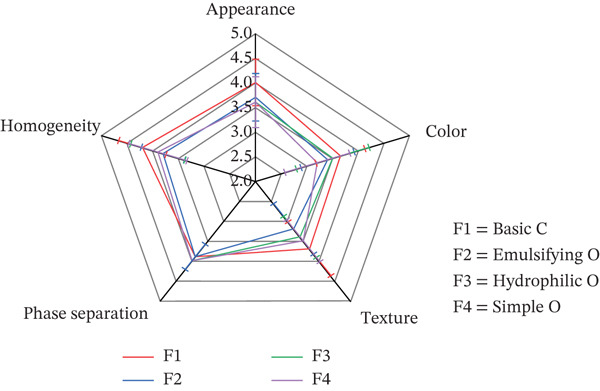
Results from the sensory evaluation performed by volunteers of semisolid formulations, expressed as the mean value (*n* = 10).

The physicochemical properties of the formulations, including pH, viscosity, and spreadability, along with key sensory attributes such as texture, homogeneity, and color, are summarized in Table 8. To visually compare the sensory profiles, a radar chart (Figure 4) was constructed using the mean scores from the panel evaluation.

**Table 8:** Physicochemical characteristics of *A*. *aspera* topical formulations (*n* = 3).

Property	Simple ointment	Emulsifying ointment	Hydrophilic ointment	Basic cream
Texture	Smooth	Smooth	Smooth	Very smooth
Homogeneity	Homogenous	Homogenous	Homogenous	Homogenous
Thermal cycle	Stable	Stable	Stable	Stable
Phase separation	No	No	No	No
Color	Dark green	Green	Dark green	Light green
Centrifugation	Stable	Stable	Stable	Stable
Spreadability (cm)	40.73 ± 0.64	37.23 ± 0.25	40.5 ± 0.5	60.5 ± 0.5
pH	7.195 ± 0.015	7.166 ± 0.03	7.290 ± 0.010	7.570 ± 0.173

The result showed that the formulations had good appearance, were homogenous, and were stable to centrifugation and thermal cycle test. The formulations exhibited neither visible change in appearance nor stability problem due to the thermal cycle test, and neither visible phase separation nor solid sedimentation was observed due to centrifugation.

The mean sensory ratings for all four product formulations across the five assessed attributes are presented in Table 9 and Figure 4. The linear mixed model analysis revealed that there were no statistically significant differences between the products for any of the sensory attributes. As illustrated in Figure 4, the mean scores for all products were closely clustered for each attribute.

**Table 9:** Mean (±SD) sensory ratings of the tested formulations.

Attributes	Basic cream	Emulsifying ointment	Hydrophilic ointment	Simple ointment
Appearance	4.0 ± 0.47	3.7 ± 0.48	3.6 ± 0.516	3.6 ± 0.5163
Color	3.65 ± 0.474	3.4 ± 0.516	3.5 ± 0.70	3.2 ± 0.63
Texture	3.7 ± 0.674	3.2 ± 0.632	3.4 ± 0.163	3.5 ± 0.527
Phase separation	3.9 ± 0.316	3.9 ± 0.316	4 ± 0	4 ± 0
Homogeneity	4.2 ± 0.46	3.8 ± 0.42	3.9 ± 0.527	3.9 ± 0.56

*Note:* Values are expressed as mean ± SEM (*n* = 3). Analysis was carried out by SPSS Version 26 using a linear mixed model. Rating is based on a 5‐point intensity scale (1 = *poor*, 5 = *excellent*).

### 4.8. Spreadability

Table 8 shows the spreading values, that is, diameters observed for the formulations, after 1 min. The values refer to the extent to which the formulations readily spread on the application surface by applying a small amount of shear. Results indicated that our semisolid preparations had differences in spreading diameters, where the higher diameter was observed by the basic cream.

### 4.9. Viscosity Measurement

Viscosity values for the semisolid preparations are shown in Figure 3. All products had a pseudoplastic behavior. The simple and emulsifying ointments had a similar viscosity curve, while basic cream and hydrophilic ointment had a lower initial viscosity.

### 4.10. Accelerated Stability Study

The accelerated stability study is presented in Table 10, and it revealed that all formulations remained physically stable, with no visible changes in physical appearance throughout the 3‐month period, with the basic cream retaining a light green color and the emulsifying ointment a green color. No signs of phase separation or discoloration were observed. The pH of the basic cream ranged from 7.57 ± 0.17 to 7.52 ± 0.14, while the emulsifying ointment ranged from 7.17 ± 0.03 to 7.28 ± 0.04, showing only minor changes over time.

**Table 10:** Stability parameters of *A. aspera* formulations stored at 40°C/75% RH for 3 months.

Formulation	Time (months)	Appearance	pH (*m* *e* *a* *n* + *S* *D*)	Viscosity (cPs, *m* *e* *a* *n* ± *S* *D*)		Spreadability (mm, *m* *e* *a* *n* + *S* *D*)	Zone of inhibition (mm, *m* *e* *a* *n* + *S* *D*)
				rpm	cPs		
Basic cream	0	Homogeneous, light green	7.57 ± 0.17	12	1200.67 ± 0.5	60.5 ± 0.5	19.00 ± 0.58
				20	800.3 ± 0.5		
				30	671.33 ± 1.5		
				50	519 ± 1		
	1	Homogeneous, light green	7.55 ± 0.12	12	1200.67 ± 2.5	60.2 ± 0.6	18.90 ± 0.50
				20	800 ± 1		
				30	669.33 ± 1.15		
				50	520.3 ± 0.57		
	2	Homogeneous, light green	7.53 ± 0.15	12	1199.3 ± 1.15	59.9 ± 0.4	18.80 ± 0.55
				20	801.67 ± 3.51		
				30	667.67 ± 0.57		
				50	518.67 ± 0.57		
	3	Homogeneous, light green	7.52 ± 0.14	12	1198.67 ± 1.15	59.8 ± 0.7	18.70 ± 0.52
				20	800 ± 2.64		
				30	666.67 ± 4.16		
				50	5219.33 ± 0.577		
Emulsifying ointment	0	Homogeneous, green	7.17 ± 0.03	12	4300 ± 2	37.2 ± 0.3	16.67 ± 0.33
				20	3202 ± 2.65		
				30	2670 ± 2		
				50	1399.67 ± 1.5		
	1	Homogeneous, green	7.22 ± 0.05	12	4301.67 ± 3.51	37.0 ± 0.4	16.20 ± 0.41
				20	3199 ± 1.73		
				30	2680.33 ± 0.57		
				50	1398 ± 2.64		
	2	Homogeneous, green	7.28 ± 0.04	12	4297.33 ± 2.08	36.8 ± 0.5	15.90 ± 0.38
				20	3198 ± 1		
				30	2667.67 ± 2.51		
				50	1397.67 ± 2.51		
	3	Homogeneous, green	7.21 ± 0.06	12	4298.67 ± 0.57	36.9 ± 0.4	15.80 ± 0.45
				20	3198.67 ± 0.57		
				30	2667.67 ± 0.57		
				50	1398.33 ± 1.52		

Viscosity measurements indicated shear‐thinning behavior. The basic cream had lower viscosity values (1200–520 cPs) compared to the emulsifying ointment (4300–1400 cPs). Viscosity remained relatively stable over 3 months. Spreadability of the basic cream (60.5 ± 0.5 to 59.8 ± 0.7 mm) was higher than that of the emulsifying ointment (37.2 ± 0.3 to 36.9 ± 0.4 mm), with a slight decrease over time.

The zone of inhibition for the basic cream ranged from 19.00 ± 0.58 to 18.70 ± 0.52 mm, while the emulsifying ointment ranged from 16.67 ± 0.33 to 15.80 ± 0.45 mm, indicating antimicrobial activity for both formulations.

## 5. Discussion

Infectious diseases are responsible for millions of deaths globally, with antibiotic resistance (AMR) ranking among the Top 10 health threats. Bacterial resistance to antibiotics has become an urgent public health and socioeconomic crisis, comprising modern medicine′s reliance on effective antimicrobial treatments. Alarmingly, high rates of resistant infections have been documented across all WHO regions [9]. Given that many bacterial strains now resist most newly developed drugs, alternative and complementary therapies could play a significant role in combating this challenge. Therefore, this study is aimed at developing and evaluating a semisolid preparation derived from a local plant as a potential therapeutic option.

The present study evaluated the antibacterial activity of 70% ethanol extracts from both fresh and dried *A. aspera* leaves against various bacterial strains. The results demonstrated significant antibacterial activity, with fresh extracts generally exhibiting stronger inhibition than dried extracts, particularly against *S. aureus*. This finding aligns with previous studies [46], which reported higher antibacterial efficacy in fresh plant materials. However, contrasting reports [47, 48] suggest that drying may enhance activity in some cases, indicating that the effect of plant state on antibacterial properties may be species‐specific.

The variation in antibacterial efficacy between fresh and dried extracts could be attributed to multiple factors, including the preservation of bioactive compounds [49], genetic and developmental differences in plant materials [17], environmental influences [50], and potential structural changes in phytochemicals during drying [51]. Unlike earlier studies that mainly assessed crude extracts of dry powders, our work advances by using both fresh and dry leaf parts and fractionating the hydroalcoholic extracts to pinpoint the most active fraction using solvents of varying polarity indices.

The greater susceptibility of Gram‐positive bacteria (*S. aureus*) compared to Gram‐negative bacteria (*E. coli* and *P. aeruginosa*) in this study has been widely documented. This trend was similarly observed by Qadir et al. [52] and Mishra [53], who reported that *A*. *aspera* extracts exhibited lower activity against Gram‐negative strains. This difference is often attributed to the complex outer membrane of Gram‐negative bacteria, which acts as a permeability barrier to many antimicrobial agents [17]. Additionally, *E. coli* was reported to be one of the most resistant bacteria globally [54], including Ethiopia [11], further supporting our findings.

The strong inhibitory effect against *S. aureus* is particularly noteworthy, as this pathogen is a major cause of skin infections [55]. This supports the traditional use of *A*. *aspera* in wound treatment and suggests its potential as a natural antimicrobial agent. The antibacterial properties may be linked to phenolic compounds such as rutin, which interact with bacterial cell walls through hydrophobic and hydrogen bonding [17].

The fractionation of *A*. *aspera* extracts using solvents of increasing polarity yielded distinct antibacterial activities, with the chloroform fraction emerging as the most potent against *S. aureus*. Notably, this fraction exhibited significantly higher inhibition zones (*p* < 0.05 based on one‐way ANOVA with Tukey′s post hoc test) compared to both the aqueous and *n*‐hexane fractions, even surpassing the activity of the original hydroalcoholic crude extract at equivalent concentrations. The enhanced efficacy of the chloroform fraction may be attributed to its ability to solubilize moderately polar bioactive compounds, such as alkaloids and certain phenolic compounds [56], which are known for their antimicrobial properties.

Similar to the present study, Habtamu and Mekonnen [57] obtained analogous results, with the chloroform extract demonstrating higher antibacterial activity against *S*. *aureus* compared to the hydroalcoholic extracts at the same dose (200 mg/mL). When comparing our results to the study by Habtamu and Mekonnen [57], it should be noted that there is a substantial difference in the results (16.50 ± 0.76 vs. 11.12 ± 0.06), which could be attributed to the state of the plant material (fresh vs. dry).

Conversely, Londonkar [58] contradicted these findings, noting that polar extracts, specifically methanol extracts, exhibited greater activity against *S*. *aureus* than their chloroform and petroleum ether counterparts. The disparity in activity might be attributed to variations in the extraction method and the antibacterial testing procedure employed. Londonkar utilized infusion for extraction and agar well diffusion for antibacterial activity testing. Infusion, due to heat‐induced solvent evaporation, may impact extraction efficiency, and the polar nature of the agar medium in agar well diffusion could impede the diffusion of nonpolar constituents. Such methodological variations highlight the need for standardized extraction protocols and assay methods. Consequently, more robust and quantitative tests such as MIC are imperative for a comprehensive assessment of the initial antibacterial activities of fractions.

The MIC results provide crucial quantitative validation of the chloroform fraction′s antibacterial potency, with values of 3.125 mg/mL against both *S*. *aureus* and *P*. *aeruginosa*, confirming the efficacy observed in preliminary screening. This quantitative result aligns with the initial findings, where the chloroform fraction showed the largest inhibition zones, particularly against *S*. *aureus*.

The significant antibacterial activity observed in the chloroform fraction (MIC = 3.125 mg/mL against *S. aureus*) most probably arises from its selective extraction of midpolarity bioactive compounds from *A. aspera*. Current evidence suggests that membrane‐disruptive saponins, particularly oleanolic acid derivatives, may permeabilize bacterial cell walls through cholesterol complexation [17], which would explain the fraction′s strong activity against Gram‐positive pathogens. This effect could potentially be enhanced by alkaloids such as achyranthine, which are known to inhibit bacterial topoisomerases and cell wall biosynthesis in other systems [16]. Simultaneously, phenolic constituents like chlorogenic acid might contribute to the observed activity by potentially interfering with quorum sensing and biofilm formation (P. [19]). The apparent superiority of the chloroform fraction over polar and nonpolar extracts suggests that these phytochemicals may be optimally concentrated by this solvent system, though further characterization is needed to confirm this hypothesis.

When compared to previous studies, our MIC results present some notable variations. The current MIC value was significantly lower than the 12.25 mg/mL reported by Habtamu and Mekonnen [57] for the same bacterial strains, suggesting that our extraction method may have been more effective at concentrating bioactive compounds. However, our MIC was higher than values reported by Ndhlala et al. [17] (0.39–1.56 mg/mL) and Mishra et al. [19] (0.3125 mg/mL), potentially due to differences in plant chemotypes, collection locations, or extraction methodologies.

Despite these variations, the MIC value falls well within the pharmacologically relevant range, as defined by Van Vuuren and Holl [59], who consider MICs below 8 mg/mL as promising for crude plant extracts, given their complex composition.

More importantly, this fraction was systematically incorporated into a wide range of semisolid bases commonly used in topical preparations, allowing us to evaluate and compare their in vitro release and antibacterial performance. This integration of phytochemical activity with formulation science provides direct evidence of how base composition and rheological behavior influence therapeutic outcomes, representing a significant advancement beyond previous antibacterial studies of *A*. *aspera*. The evaluation of eight semisolid formulations revealed significant variations in antibacterial activity against *S*. *aureus*, which can be attributed to differences in base composition and drug release properties.

The spectrum of the pure *A. aspera* fraction showed characteristic peaks at 3435 cm^−1^ (O–H), 2920 and 2850 cm^−1^ (C–H), and multiple fingerprint peaks between 1800 and 600 cm^−1^. However, in both the basic cream and emulsifying ointment formulations (Figures 1 and 2), several of these fingerprint peaks (e.g., with the range of 930–1500 cm^−1^) were notably absent.

While this deviation from an additive spectrum could initially suggest incompatibility, it is critical to note that no new peaks appeared in either formulation. The absence of new peaks, therefore, may suggest that the observed changes could be due to physical factors, specifically dilution or spectral masking, rather than chemical degradation. This confirms that the *A. aspera* fraction remains chemically stable within both formulations.

Among the tested formulations, basic cream demonstrated the highest antibacterial activity, followed by emulsifying ointment, simple ointment, and hydrophilic ointment. These differences likely arise from variations in excipient composition, particularly creams and ointments containing emulsifying agents like sodium lauryl sulfate, which exhibited superior activity compared to gel‐based formulations. These findings align with previous studies suggesting that emulsifiers enhance drug release by improving solubility and diffusion of active compounds (Jankowski, Dyja, and Sarecka‐Hujar 2017; [22]).

The superior performance of basic cream and emulsifying ointment may be explained by their shared composition of emulsifying wax (90% cetostearyl alcohol and 10% sodium lauryl sulfate), which facilitates the partitioning of both hydrophilic and hydrophobic active ingredients. This observation is consistent with earlier reports ([24, 60]), where formulations containing emulsifiers demonstrated enhanced antimicrobial activity due to improved drug release kinetics.

The better antibacterial performance of basic cream among the tested formulations supports findings by Bakker et al. [3], who similarly reported enhanced activity in cream‐based formulations compared to ointments. This phenomenon appears directly related to the fundamental physicochemical properties of cream bases, where their biphasic nature and incorporation of emulsifiers like sodium lauryl sulfate facilitate more efficient partitioning and release of active compounds compared to single‐phase systems. The presence of both aqueous and lipid phases in creams likely creates optimal conditions for solubilizing diverse phytochemical constituents while maintaining favorable release kinetics.

In contrast, gel bases, which are composed of synthetic polymers like carbomers or cellulose derivatives, likely retained active compounds due to their high affinity for APIs, resulting in poor diffusion and reduced antibacterial efficacy [23]. This discrepancy underscores the critical role of base selection in optimizing topical drug delivery.

The release of active compounds and subsequent antibacterial efficacy were demonstrably influenced by the formulation base. The superior performance of lipophilic ointments over hydrophilic gels suggests that a lower affinity between the vehicle and the middle‐polar extract promotes diffusion into the aqueous agar medium. However, interpreting agar diffusion results requires caution: The inhibition zone is a function of both the antimicrobial potency and the diffusibility of the formulation, which is affected by its viscosity, hydrophobicity, and emulsifying properties.

Therefore, while the basic cream was identified as the optimal formulation in vitro, its translational potential requires further investigation. In vivo performance is contingent upon additional variables like skin barrier function and application site [21]. To fully establish clinical relevance, subsequent studies should focus on in vivo models and expand antimicrobial testing to include critical pathogens such as MRSA.

The physical stability of topical formulations under various stress conditions is a critical factor in ensuring their quality and efficacy during storage and transportation. In this study, the effects of centrifugation and thermal cycling on the stability of the formulations were evaluated to simulate harsh handling and climatic variations.

Centrifugation was employed to assess the formulations′ resistance to phase separation and sedimentation under mechanical stress. The results demonstrated that none of the tested formulations exhibited visible phase separation or solid sedimentation after centrifugation. This suggests that the formulations possess strong physical stability and can likely withstand vigorous handling conditions, such as those encountered during lengthy transportation to remote areas. The absence of instability under high centrifugal force indicates that the formulations maintain their structural integrity, which is essential for ensuring consistent drug delivery upon application.

Additionally, thermal cycle testing was conducted to evaluate the formulations′ stability under fluctuating temperatures, simulating real‐world conditions where products may be exposed to varying climatic temperatures between room temperature (25°C) and higher temperatures (50°C). The tested formulations showed no visible changes in appearance or signs of instability after undergoing thermal cycling. This indicates that the formulations are robust enough to endure temperature variations without compromising their physical properties. Such stability is particularly important for topical products, as exposure to temperature fluctuations during storage and distribution could otherwise lead to changes in viscosity, phase separation, or active ingredient degradation.

Accelerated 3‐month stability studies were conducted for both basic cream and emulsifying ointment. Formulations were stored under controlled conditions and evaluated monthly for appearance, pH, viscosity, spreadability, and antibacterial activity. Both formulations remained homogeneous, with the basic cream retaining a light green color and the emulsifying ointment a green color throughout the study. The pH of the basic cream decreased slightly from 7.57 ± 0.17 to 7.52 ± 0.14, while the emulsifying ointment ranged from 7.17 ± 0.03 to 7.28 ± 0.04. The initial viscosity and spreadability values varied minimally but were not significant over time. Despite these minor changes, both formulations maintained satisfactory antibacterial activity against *S. aureus*, confirming that the extract remained stable and functional in the semisolid bases during the 3‐month accelerated study.

The viscosity and spreadability of topical formulations are critical factors influencing drug release, ease of application, and therapeutic efficacy. The results of this study demonstrated that the tested formulations exhibited pseudoplastic flow behavior, as evidenced by the decrease in viscosity with increasing shear rate (from 12 to 50 rpm). This rheological property is desirable for topical products, as it ensures that the formulation maintains sufficient viscosity at rest (preventing dripping or phase separation) while becoming less viscous upon shear stress (e.g., during spreading), facilitating smooth application.

Among the tested formulations, the basic cream and hydrophilic ointment exhibited the lowest viscosities. This characteristic likely contributed to the enhanced drug release observed in the basic cream, as lower viscosity reduces resistance to drug diffusion, allowing for faster and more efficient transfer of the API from the vehicle into the hydrophilic agar medium. This finding suggests that formulations with lower viscosity may be advantageous when rapid drug release is desired, though a balance must be struck to ensure adequate residence time at the application site.

Spreadability is another crucial parameter for semisolid formulations, as it determines how easily and uniformly the product can be applied to the skin. The results indicated that the basic cream had the highest spreadability, correlating with its lower viscosity. A more spreadable formulation ensures that a consistent and appropriate dose is delivered to the target area, improving patient compliance and therapeutic outcomes. The inverse relationship between viscosity and spreadability observed in this study aligns with rheological principles where formulations with lower structural resistance (lower viscosity) spread more readily under applied shear force (e.g., fingertip pressure).

The result of the acute dermal toxicity test showed that the 10% semisolid preparations showed no sign of inflammation, irritation, or redness, which suggested the safety profile of the extract along with the excipients used. This supports the traditional claim of the plant for wound healing.

The present study formulated the extract at a single concentration of 10% *w*/*w*. While this concentration was justified based on existing literature [29, 30] and the extract′s MIC value, a full dose–response optimization within the vehicle was not performed due to limitations in extract yield. The efficacy demonstrated at this concentration confirms its potential as a topical agent; however, future studies are necessary to determine the minimum effective and most economical formulation concentration. This work establishes a foundation for such optimization.

## 6. Conclusion

In conclusion, this study demonstrated that chloroform fractions of the leaves of *A. aspera* can be formulated into safe and effective topical preparations. Among the tested formulations, the basic cream showed superior antibacterial activity against *S. aureus* while also exhibiting desirable physicochemical properties and skin tolerability. The 3‐month accelerated stability study further confirmed that both the basic cream and emulsifying ointment maintained their appearance, pH, viscosity, spreadability, and antibacterial efficacy, highlighting their physical and chemical stability. Additionally, compatibility studies (FTIR analysis) indicated no chemical interactions between the extract and formulation excipients, confirming the stability of the active compounds within the semisolid bases. These findings highlight the potential of *A*. *aspera* as a natural and sustainable alternative for managing bacterial skin infections. However, this work was limited to in vitro assays with small‐scale formulations. Future research should focus on in vivo assessments, dose optimization, bioactive compound characterization, and long‐term stability studies to fully establish its clinical applicability.

## Funding

The study was funded by Mekelle University, 10.13039/501100009402.

## Conflicts of Interest

The authors declare no conflicts of interest.

## Data Availability

All the original data is available upon request from the corresponding author.
